# Pleomorphic Xanthoastrocytoma: a single institution retrospective analysis and a review of the literature

**DOI:** 10.1007/s11547-022-01531-3

**Published:** 2022-08-11

**Authors:** Beatrice Detti, Silvia Scoccianti, Virginia Maragna, Sara Lucidi, Michele Ganovelli, Maria Ausilia Teriaca, Saverio Caini, Isacco Desideri, Benedetta Agresti, Daniela Greto, Anna Maria Buccoliero, Alessandro Della Puppa, Iacopo Sardi, Lorenzo Livi

**Affiliations:** 1 Radiation Oncology Unit, University of Florence - Azienda Ospedaliero-Universitaria Careggi, Largo Brambilla 3, Florence, Italy; 2 Epidemiology of Risk Factors and Lifestyles, Institute for Study, Prevention, and Oncology Network (ISPRO), Florence, Italy; 3 Pathology Unit, Children’s Hospital A. Meyer, University of Florence, Florence, Italy; 4 Department of Neurosurgery, Careggi Hospital, University of Florence, Florence, Italy; 5 Neuro-Oncology Unit, Department of Pediatric Oncology, Meyer Children’s Hospital, Florence, Italy

**Keywords:** Pleomorphic Xanthoastrocytoma, Radiotherapy, Brain tumor

## Abstract

**Background:**

Pleomorphic xanthoastrocytoma (PXA) is a rare low-grade brain tumor. To date, limited studies have analyzed factors affecting survival outcomes and defined the therapeutic strategy. The aim of this retrospective analysis was to investigate the clinicopathologic characteristics of PXA and identify factors associated with outcomes.

**Methods:**

We retrospectively analyzed a cohort of 16 adult and children patients with PXA who underwent primary resection from 1997 to 2019, referred to our Radiation Oncology Unit and to Meyer’s Paediatric Hospital. We also reviewed the relevant literature.

**Results:**

All patients underwent primary surgical resection; 10 patients received adjuvant radiation treatment course, ranging from DTF 54 to 64 Gy; 8 of them received, in addition, concurrent adjuvant chemotherapy; 6 patients underwent only radiological follow-up. After a median follow up was 60 months: median OS was 34.9 months (95% CI 30–218), 1-year OS 87%, 5-years OS 50%, 10-years OS 50%; median PFS 24.4 months (95% CI 13–156), 1-year PFS 80%, 5-years PFS 33%, 10-years PFS 33%. A chi-square test showed a significant association between OS and recurrent disease (*p* = 0.002) and with chemotherapy adjuvant treatment (*p* = 0.049). A borderline statistical significant association was instead recognized with BRAF mutation (*p* = 0.058).

**Conclusions:**

Despite our analysis did not reveal a strong prognostic or predictive factor able to address pleomorphic xanthoastrocytoma management; however, in selected patients could be considered the addition of adjuvant radiation chemotherapy treatment after adequate neurosurgical primary resection. Furthermore, recurrent disease evidenced a detrimental impact on survival.

## Introduction

Pleomorphic xanthoastrocytoma (PXA) is a rare low-grade astrocytic tumor, accounting for < 1% of all astrocytomas with a good prognosis, exhibiting a 10-year survival of more than 70%. The World Health Organization (WHO) 2016 classification introduced the anaplastic PXA (aPXA), as a distinct entity compared to the lower grade counterpart, characterized by stimulating mitotic activity (i.e. the presence of 5 or more mitoses for 10 high-powered fields (HPF) [1], MIB1 index > 4%, higher necrosis and microvascular proliferation and in addition to a more common cerebrospinal fluid (CSF) spreading, a worse outcome.

Both de novo presentations of WHO grade III aPXA and progression of grade II PXA have been observed: about 20% of PXA may develop anaplastic features during his course. Mutation of the TERT telomerase reverse transcriptase (TERT) promoter is the second most common alteration in anaplastic PXA, after BRAF V600E mutation and CDKN2A homozygous deletion, and these genetic alterations could be related to anaplastic progression from PXA [2]. Vemurafenib, a BRAF V600 kinase inhibitor, in fact, shows promising activity in mutated gliomas, with most benefits in PXA [3]. Currently, the therapeutic strategy takes into account the surgery extent and histologic grade: despite no clear survival benefit obtained by adjuvant radiotherapy, the treatment is frequently added in case of incomplete gross resection and/or in case of anaplastic features, with or without temozolomide chemotherapy. Radiation course in range of 45–54 Gy is also the preferential salvage treatment proposed [4, 5].

Herein, we retrospectively reviewed 16 PXA cases, both of pediatric and adult age, to further clarify the natural history and prognosis of this tumor and to analyze impact of treatments on survival endpoints.

## Materials and methods

We retrospectively analyzed an unselected cohort of 16 xanthoastrocytoma patients, who underwent primary resection from 1997 to 2019, referred to our Radiation Oncology Unit, AOU Careggi, and to Meyer’s Paediatric Hospital in Florence, Italy. Patient’s baseline characteristics were summarized in Table 1.

**Table 1:** Patients characteristic

Feature	Patients	%
Sex		
M	6	37.5
F	10	62.5
Age at diagnosis		
≤ 16 years	5	31.3
> 16 years	11	68.7
Symptoms at initial presentation		
Yes	14	87.5
No	2	12.5
*Disease site*		
Left cerebral hemisphere		
Frontal lobe	3	18.7
Temporal lobe	5	31.3
Occipital lobe	1	6.3
Right cerebral hemisphere		
Temporal lobe	3	18.7
Occipital lobe	1	6.3
Parietal lobe	3	18.7
Surgical radicality		
Complete excision	15	93.8
Residual disease	1	6.2

All cases were classified in agreement with WHO CNS (Central Nervous System) 2016 Classification as pleomorphic xanthoastrocytoma (grade II) and anaplastic pleomorphic xanthoastrocytoma (grade III): anaplastic pattern was defined by 5 or more mitoses per 10 high-power fields. Presence of BRAF V600E mutation, MGMT (O6-methylguanine-DNA methyltransferase gene) promoter methylation, IDH1 (isocitrate dehydrogenase 1) mutation, ATRX (ATP-dependent helicase ATRX) mutation, and CD34 marker presence were reported.

After surgery, patients were addressed to active surveillance or to adjuvant radiation therapy treatment alone or in combination with chemotherapy. Data on doses, number of cycles, and treatment tolerance were collected and adverse effects graduated, according to Common Terminology Criteria for Adverse Events (CTCAE), version 4.0 [6]. Treatment response was evaluated by periodic MRI according to Response Assessment in Neuro-Oncology Criteria (RANO) Criteria [7]: every three months imaging for the first 2 years from primary treatment, prolonging to four to six months in the subsequent 3 years, and every 8–12 months thereafter. In case of recurrent disease, data about timing, treatments, and anatomopathological changed features were collected.

PFS was defined as the time from primary neurosurgical treatment until progression or death from any cause or to the last day of follow-up.

OS was calculated from the date of primary neurosurgical treatment to the date of the most recent follow-up or death from any cause.

PFS and OS were estimated using the Kaplan–Meier (KM) method and 95% CIs. Log-rank test was applied to detect a difference survival endpoint, with null hypothesis of no survival differences between groups for Overall Survival (OS) and of no time to progression differences between groups for Progression Free Survival (PFS). A p-value ≤ 0.05 was considered statistically significant to reject null hypothesis. Cox regression analysis was also performed to assess survival endpoints. Fisher’s exact test was also applied to analyse if statistical significant differences between defined class frequencies exist.

## Results

From January 1997 till January 2019, 16 xanthoastrocytoma patients were treated at our Radiation Oncology Unit. Median follow-up was 60 months (range 4–218 months).

Median age at presentation was 31.3 years (range 6–69 years). The most frequent symptoms of disease appearance were focal neurological disorders (56.3%), frequently patients described long history of months with headache (31.3% of the entire cohort), only three patients (18.8%) onset was characterized by seizures. See Table 1 for details.

Median lesion dimensions were 3.8 cm, ranging from 1.9 to 7 cm. Localization of PXA was in the left cerebral hemisphere in 56.3% of cases, and in the right one in 43.8%.

Anatomopathological features confirmed by an independent anatomopathological revision were reported in Table 2.

**Table 2:** Pathological features

Feature	Patients	%
Xanthoastrocytoma grade		
Grade II	4	25
Grade III	12	75
MGMT status		
Unknown	5	31.25
Metilated	6	37.5
Unmetilated	5	31.25
IDH 1 mutation		
Unknown	2	12.5
Mutated	0	0
Not Mutated	14	87.5
BRAF mutation		
Unknown	1	6.25
V600E mutated	5	31.25
Not Mutated	10	62.5
ATRX mutation		
Unknown	3	18.75%
Mutated	13	81.25%
Not Mutated	0	0%
CD34 marker		
Positive	9	56.25
Negative	6	37.5
Unknown	1	6.25

Fourteen patients underwent an early postoperative contrast-enhanced MRI while 2 patients, treated in 1998 and 2003 respectively, underwent only a postoperative contrast-enhanced CT, to define surgical radicality. Postoperative MRI identified in one patient residual disease and was used to help the planning of radiation therapy treatment.

Ten patients received an adjuvant radiation treatment course, ranging from DTF 54 to 64 Gy (median dose 60 Gy) with a single daily fraction ranging from 2 to 1.8 Gy, delivered on five consecutive days a week. 8 of them received concurrent adjuvant chemotherapy: all patients were treated with temozolomide 75 mg/mq body surface daily during entire radiation course, and one of them received a doublet containing temozolomide and vinorelbine 30 mg/mq body surface weekly. Number of adjuvant chemotherapy cycles with the same agent ranged from 9 to 25 (median number of cycles 19.6).

Six patients underwent only follow-up, and 5 of them relapsed after a median time of 16.5 months (average 35.5 months, range 153–8 months): 2 patients were eligible for re-surgery and one patient received radiation treatment course. The other two patients were not eligible for active therapies due to clinical performance status. Anatomopathological analysis after re-surgery showed an evolution of anaplastic features.

**Fig. 1:**
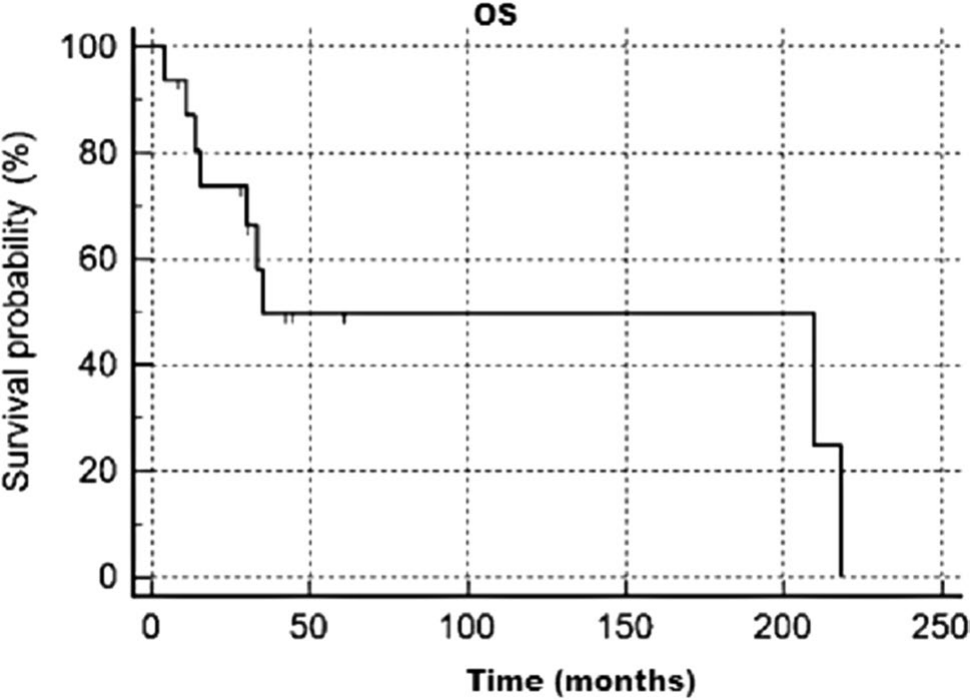
Overall survival analysis

**Fig. 2:**
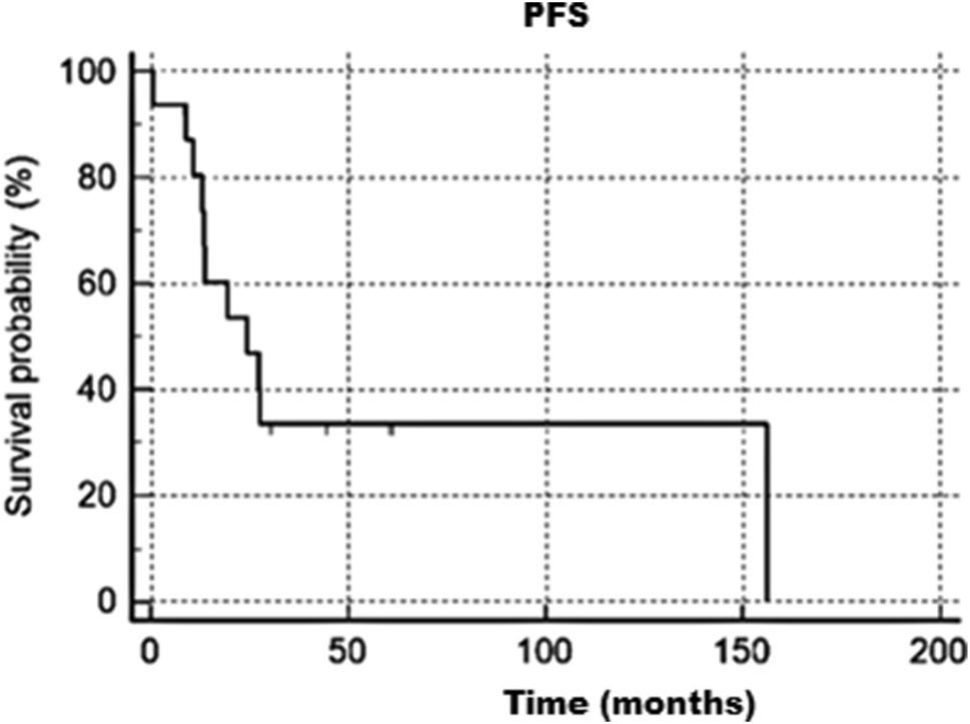
Progression free survival analysis

Median OS was 34.9 months (95% CI 30–218), 1-year OS 87%, 5-years OS 50%, 10-years OS 50%; survival rate at 18 years was 24.9%. Median PFS 24.4 months (95% CI 13–156), 1-year PFS 80%, 5-years PFS 33%, 10-years PFS 33%. Kaplan Meier OS and PFS graphs are reported below in Figs. 1 and 2.

**Fig. 3:**
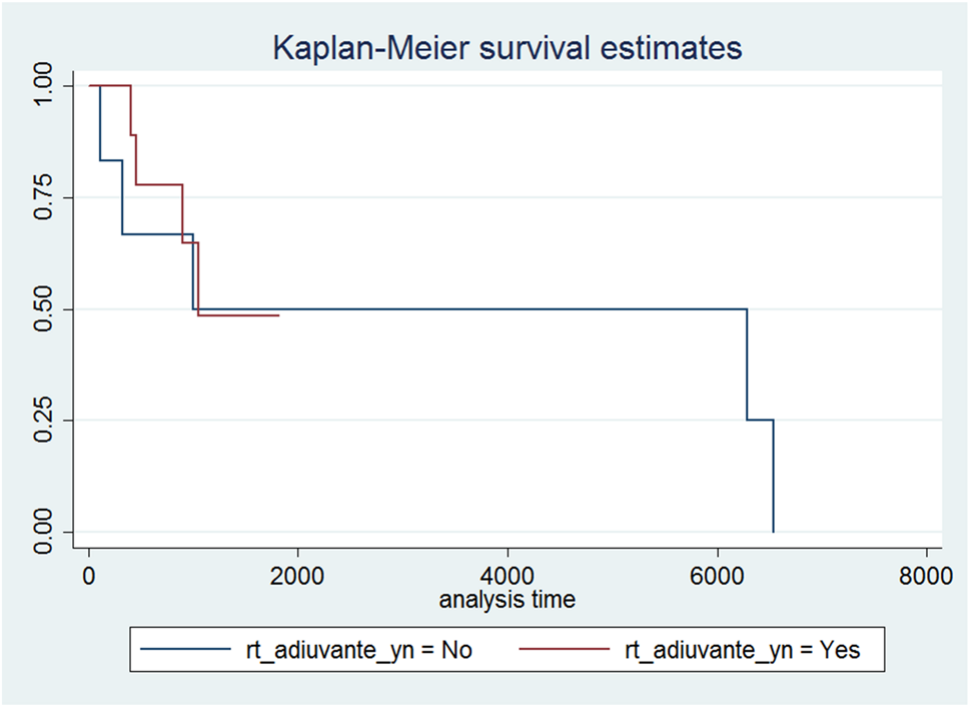
OS by adjuvant RT yes vs. no

**Fig. 4:**
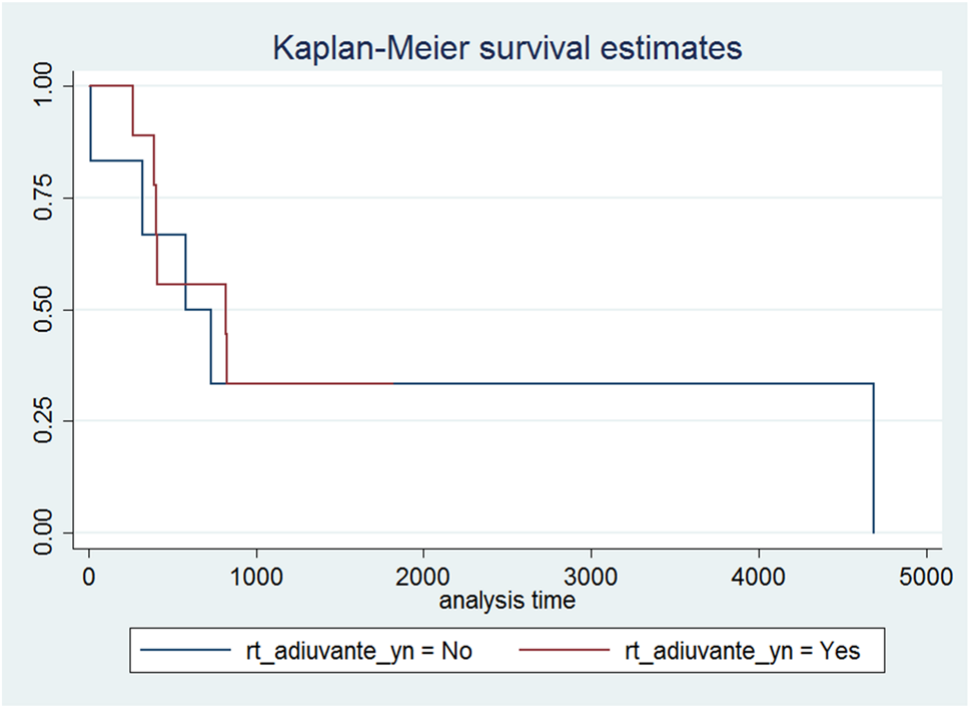
PFS by adjuvant RT yes vs. no

OS e PFS did not significantly differ between patients that underwent vs. did not undergo adjuvant radiotherapy (*p*-value 0.815 and 0.819, respectively), see Figs. 3 and 4.

At KM univariate analysis no one of variables as sex, age, neuroanatomical localization, histological grade, adjuvant chemotherapy and radiotherapy, MGMT methylation status, IDH mutation, BRAF mutation and mib1 value resulted significative in terms of PFS and OS.

A Fisher’s exact test of independence showed that there was a nearly significant association between tumor location and PFS in favor of temporal lobe site (*p* = 0.09) but at KM analysis median PFS was 27.3 months (95% CI 13–27.3) in temporal location and 13.7 months (95% CI 13.5–27.6) for other localizations (*p* = 0.46). OS showed an association with recurrent disease (*p* = 0.005) and (nearly significant) also with chemotherapy adjuvant treatment (*p* = 0.072). A borderline statistical significant association was instead recognized with BRAF mutation (*p* = 0.089).

## Discussion

Our final results, with evidence of the detrimental impact of recurrent disease on survival, point to the focus on importance of adequate primary management of disease. This could be obtained with adequate neurosurgical resection and with adjuvant radiation treatment and adjuvant chemotherapy, in selected patients. Our analysis didn’t evidence a strong prognostic or predictive factor able to address PXA management. Case by case multidisciplinar CNS expert’s discussion could help to select patients who most benefit from more aggressive initial treatment, with global considerations about molecular emerging diagnostics, anaplastic features, radical primary surgery and age of patients.

With limitations of restricted casistic, rare disease and nature of retrospective study, with observations dating back to 1997, when molecular diagnostics were less capillary, BRAF mutation overlooks a prognostic factor.

An estimated incidence of all astrocytic tumors in Italy of 4.92/100.000 was reported in last 2015 AIRTUM register [8]: most frequently disease appears in second decade of life without sex predilection and most commonly is localized at temporal lobe, potentially spreading via CSF and frequent leptomeningeal involvement. Rarely were seen other presentation features as seen as solid lesions involving the ventricular system. Symptoms of onset are consistent with mass effect, predominantly headache, and neurological disorders ranging from focal symptoms depending on site, to epileptic seizures [9–14].

Prognosis appears to be related to mitotic index: aPXA has a 5-year OS rate of 57% and PFS of 49% and PXA has 5-year survival rates of > 75% and PFS > 60% [5].

Our cohort data, with a median age of 31.3 years (range 6–69 years) at diagnosis and temporal lobe occurrence in 50% of patients conform to the epidemiological findings; the most frequent onset symptoms recorded were long-lasting headache and focal neurological manifestations.

Literature experiences evidence as prognostic factors: a complete surgical resection, young age < 20 years [15, 16] and low histological grade [4, 17].

A recent systematic review on 325 patients, besides confirming age and extent of surgery as prognostic factors affecting PFS and OS, estimated at 5 years PFS of 51.2% and a 5 years OS of 78%. [16] Our cohort doesn’t recognize the mentioned above factors as prognostic, but the small sample size could be an explanation for results. Our results, in a smaller cohort than that mentioned, evidenced a poorer 5 years OS of 49.7 versus 78% but notably 10 years OS was superimposable at 5 years OS.

Impact of adjuvant radiation treatment is not clearly beneficial and clinical practice often relies on single centre experiences and negative prognostic factors as surgery extent and anaplastic features: 45–54 Gy radiation course is the most used regimen with or without temozolomide. In our experience chemotherapy seems to be useful at a low statistical significance level; our data confirm the grey area around beneficial adjuvant treatments.

No data actually confirms an advantage in survival outcomes; Table 3 collects the most numerous experiences in the management of pleomorphic xanthoastrocytoma. Moving on an era of personalized medicine, efforts are made to distinguish molecular disease subgroups into the same anatomopathological container [26]: constitutive activation of BRAF (rapidly accelerated fibrosarcoma kinase) is the most frequently genetic mutation in PXA (66% of cases) and fewer in aPXA (65%), affecting cellular proliferation, differentiation and survival [27]. Anatomopathological findings evidence an association between V600E mutation and temporal lobe located PXA, besides CD34 positivity and reticulin fiber formation; BRAF positivity and loss of p16 expression could be a helpful tool for differential diagnosis of PXA entity with giant-cell glioblastoma and ganglioglioma [28]. Our PXA cohort appeared to be constituted only by 25% BRAF mutated patients, but this could be related to a very high quote of untested histological samples (43.7%) because of uncommon practice in past years.

**Table 3:** Main literature

Author (year) journal	Study	Patients Characteristics	Treatment	Relapse treatment	Outcomes
J Jhon Kepe Cancer (1979) [18]	Case Reports (1948–1979)	12 pts, median age 12.4 years PXA	Surgery and adjuvant RT (50%) median dose 4366 rad VS observation	Resurgery in 25%	PFS at 5 years 75% OS nd
Giannini C. Cancer (1999) [19]	Retrospective	71 pts,median age 26 years PXA	GTR 68% STR in 32% Adjuvant RT alone in 29% and Adiuvant CT RT 12.5%	Nd	PFS at 5 years 72% PFS at 10 years 61% OS at 5 years 81% OS at 10 years 70%
Pasquale Gallo British Journal of Neurosurgery (2013) [20]	Retrospective (1990–2008)	40 pts median age 30.5 years PXA 80% aPXA 20%	Surgery 60% Adjuvant RT 17.5% Adjuvant RT and TMZ 22.5%	Nd	OS at 5 years 85% vs 40% PFS at 5 years 85% vs 30% (PXA vs aPXA) OS at 5 years 80% vs 50% PFS at 5 years 80% vs 35% (GTR vs STR)
C.M. Ida Brain Pathology (2015) [21]	Retrospective (1965–2013)	74 pts median age 21.5 years PXA 69% aPXA 31%	GTR 57% STR 41%	- RT 9.8% for PXA and 13.0% for aPXA - RS 2.0% only PXA - CHT + / − RT/RS 21.6% for PXA and 60.9% for aPXA - RT + RS 4.3% only aPXA	5 years RFS 89.4% vs 45.4% GTR vs STR 5 years OS 43.3% vs 74.4% ( aPXA vs PXA)
Prita Pradhan International Journal of Hematology-Oncology and Stem Cell Research (2018) [22]	Retrospective (2012–2016)	5 pts, mean age 22 years APAXs	Surgery	Nd	nd
JingYan Scientific RePortS (2018) [23]	Retrospective (2011–2017)	50 pts, median age 36 years, PXA 52% aPXA 48%	GTR 74% STR 20% PTR 6%	Nd	PFS at 5 years 8% vs 88% (aPXA vs PXA)
Tryggve Lundar J Neurosurg Pediatr (2019) [24]	Retrospective trial (1972–2015)	12 pts, median age 8 years PXA	Surgery followed in 8.3% by adjuvant RT 54 Gy	41,7% resurgery	OS at 20 years 88%
Supriya Mallick J Neurosci Rural Pract (2019) [16]	Meta-analysis	325 pts, median age 19 years PXA 76.6% aPXA 23.4%	GTR 56,1% STR 31.4% Adjuvant RT 27.4% adjuvant RT + CHT 14.9%, mostly TMZ	26.3% resurgery, 17.1% RT, resurgery and RT 17.2%, Surgery + RT + CT 23.7%, CT + RT 9.2%, Surgery + CT 3.9%, CT 2.6%	PFS at 2 years 68.5% PFS at 5 years 51.2% OS at 2 years 88.8% OS at 5 years 78%
Marc C. Chamberlain Journal of Neuroncology (2013) [25]	Retrospective case series	4 pts, median age 45 years BRAF V600E mutated, recurrent PXA	Vemurafenib 960 mg twice daily		Mean PFS 5 months, mean OS 8 months

*PXA*  Pleomorphic xanthoastrocytoma,* aPXA*  anaplastic xanthoastrocytoma,* OS* Overall survival,* PFS* progression free survival,* GTR* gross total resection;* STR* subtotal resection;* PTR*  partial resection;* TMZ*  Temozolomide;* RT*  radiotherapy;* PVC* procarbazine, lomustine, vincristine

V600E mutated entities show better survival with respect wild-type ones and clinically observed response to BRAF kinase inhibitors Vemurafenib and Dabrafenib. Case reports suggest effective and durable responses to target therapy and useful rechallenge treatment after discontinuation, acting the drugs with prevalent cytostatic mechanism, with reported responses up to 30 months [29–32]. Clinical phase I/II trials mostly with Dabrafenib and Trametinib combination are ongoing [33].

In our experience, data about BRAF V600E mutation add up to literature, supporting molecular subtype existence with good prognosis, even if results about correlation between genetic alteration and overall survival are statistically significant borderline. In our population none were treated with target therapies against V600E mutation, we couldn’t then conclude about its predictive role. In Italy none of anti-BRAF agents are actually approved by AIFA (Italian Agency of Pharmaceuticals).

Other molecular observations under preclinical investigation include TERT promoter alterations, which seems to be related to anaplastic progression, [2, 34, 35] apparently low rate of IDH1/IDH2 mutations and MGMT promoter hypermethylation, with differential diagnosis implications with Glioblastoma Multiforme (GBM) [36]. The results observed in our study are in line with an epidemiological, clinical and histological point of view with what has already been observed in the literature. Even in our study group, the onset was mainly at a young age, surgery was the first choice treatment followed by radiation therapy in combination or not with chemotherapy.

Our study includes rare disease patients treated by specialists with high volume and high expertise in CNS neoplasms: every single case was discussed in a weekly multidisciplinary meeting to choose the best option for the patient, in light of surgical, radiological anatomopathological and radio-oncological findings.

Limitations of the current study are due to the retrospective nature, small sample size, heterogeneity of the characteristics of the population, furthermore the treatment at relapse did not include the new BRAF inhibitors, which recently have demonstrated activity in clinical observations.

## Conclusion

PXA is a rare disease that occurs mainly in young adults. On the basis of data in the literature, younger patients (< 20 years), patients who undergo a GTR, and patients with a lower grade tumor have a better outcome. Grade III and incompletely resected tumors, adjuvant radiation or a combination of both radiotherapy and chemotherapy should be delivered taking into account risk factors, while the role of adjuvant therapy is debatable. The molecular characteristics should be performed to identify patients with different clinical behavior, they could influence not only prognosis but also therapeutic management. Target therapies are the new perspectives towards which studies are directed.
